# Supplementary material for: ZebraShare: a new venue for rapid dissemination of zebrafish mutant data
Source: PeerJ. 2021 Apr 13;9:e11007. doi: 10.7717/peerj.11007 (PMC8051354; doi:10.7717/peerj.11007)

Samples: 22672
Bases: 614
Average spacing: 37



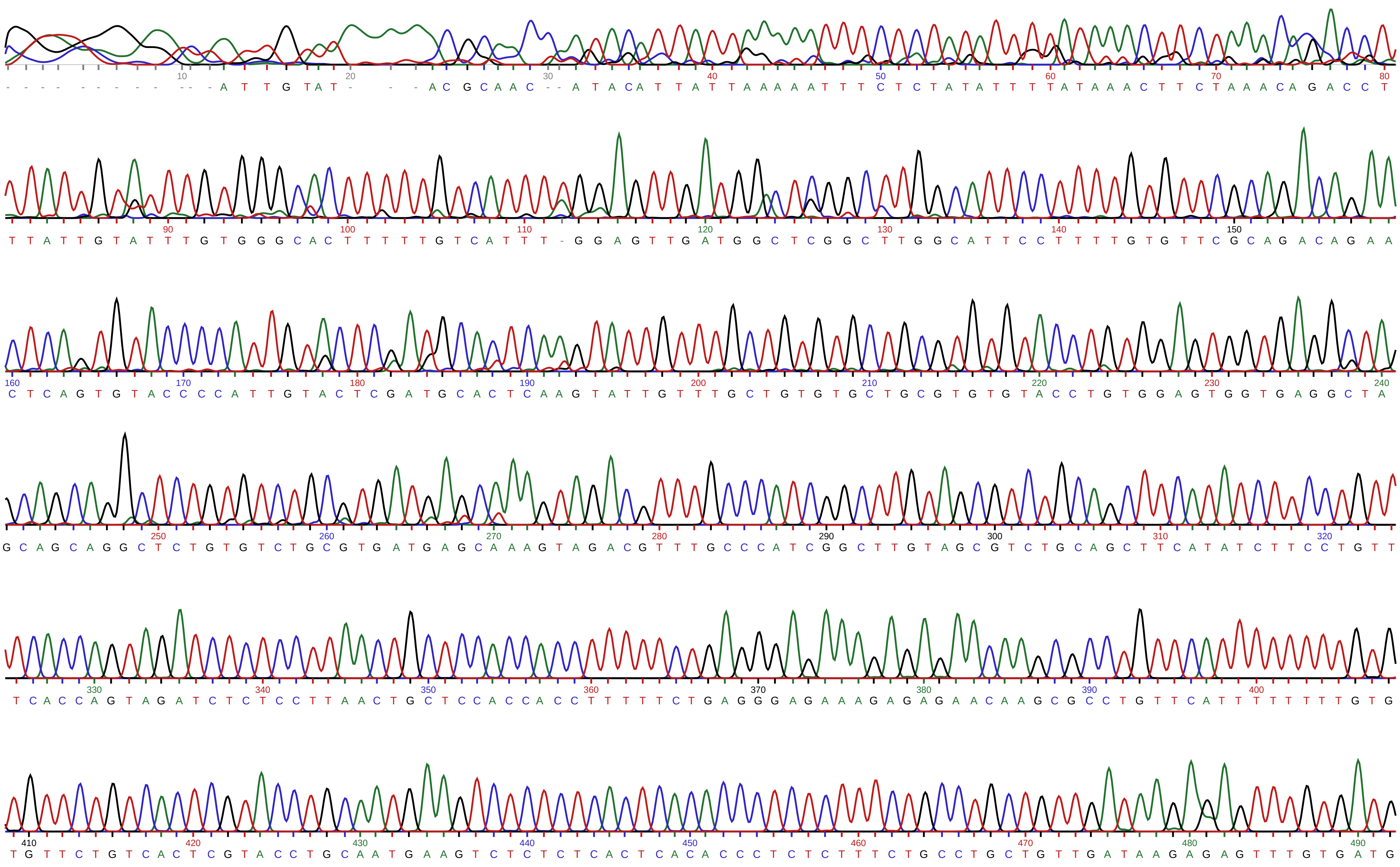

Samples: 22672
Bases: 614
Average spacing: 37



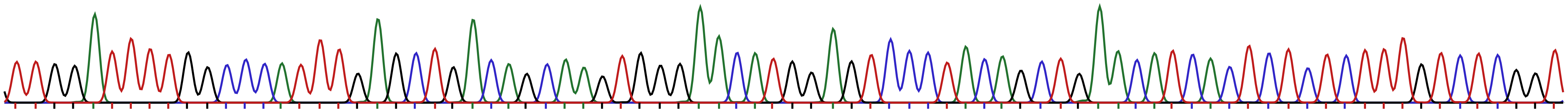

500 510 520 530 540 550 560 570

T T G G A T T T T G G C C C A T T T G A G C T G A C A G C A A G T G G G A A C A T G G A G T C C C T A C A G C T G A A C A T C A C T C T C T C T T T G T C T C G G T

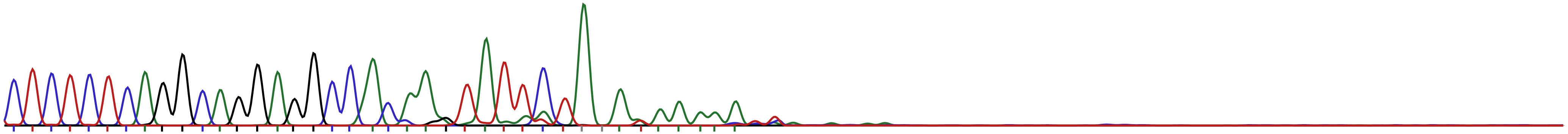

580 590 600 610

C T C T C T C A G G C A G G A G G C C A C A A G T A T T C T - - A T A A A A A

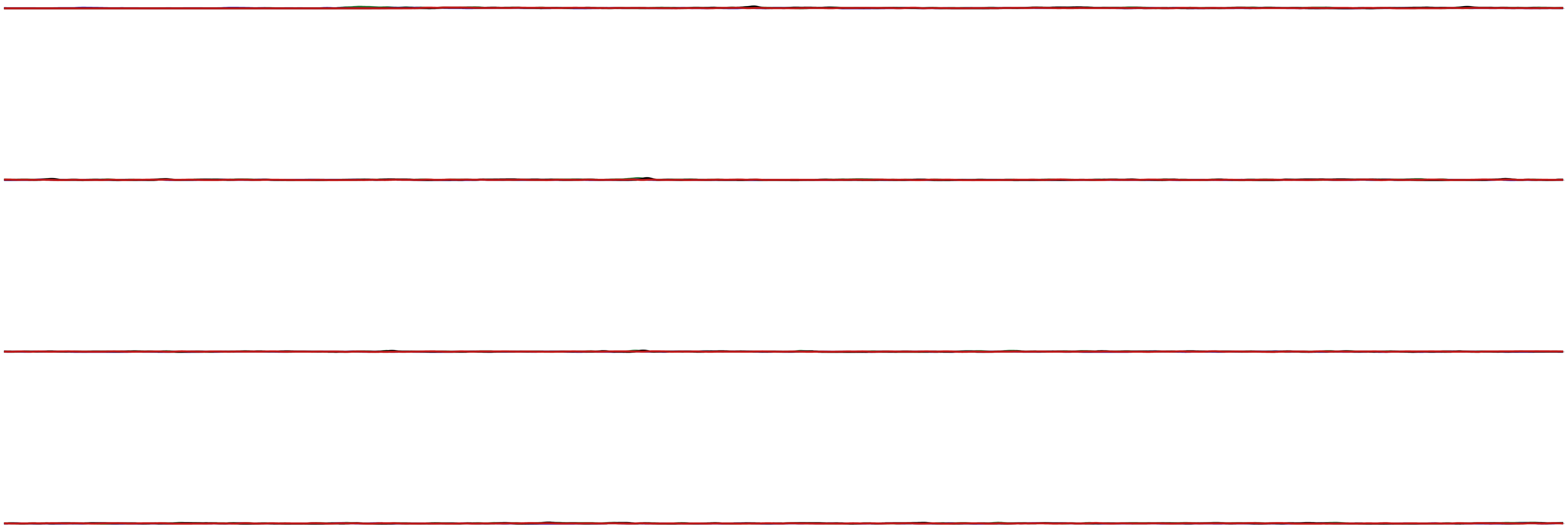

| | |
|---|---|
| Samples: | 22672 |
| Bases: | 614 |
| Average spacing: | 37 |

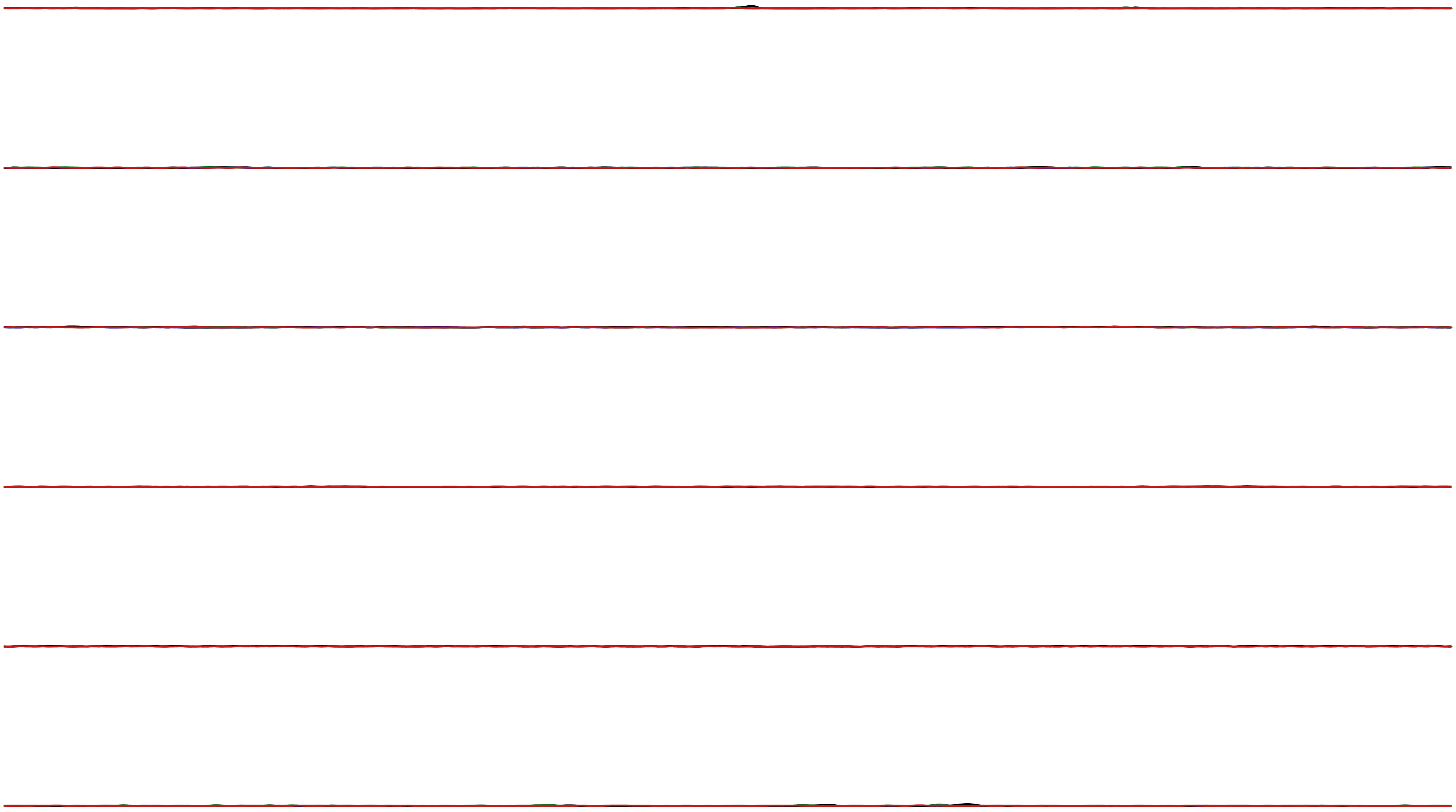

| | |
|---|---|
| Samples: | 22672 |
| Bases: | 614 |
| Average spacing: | 37 |



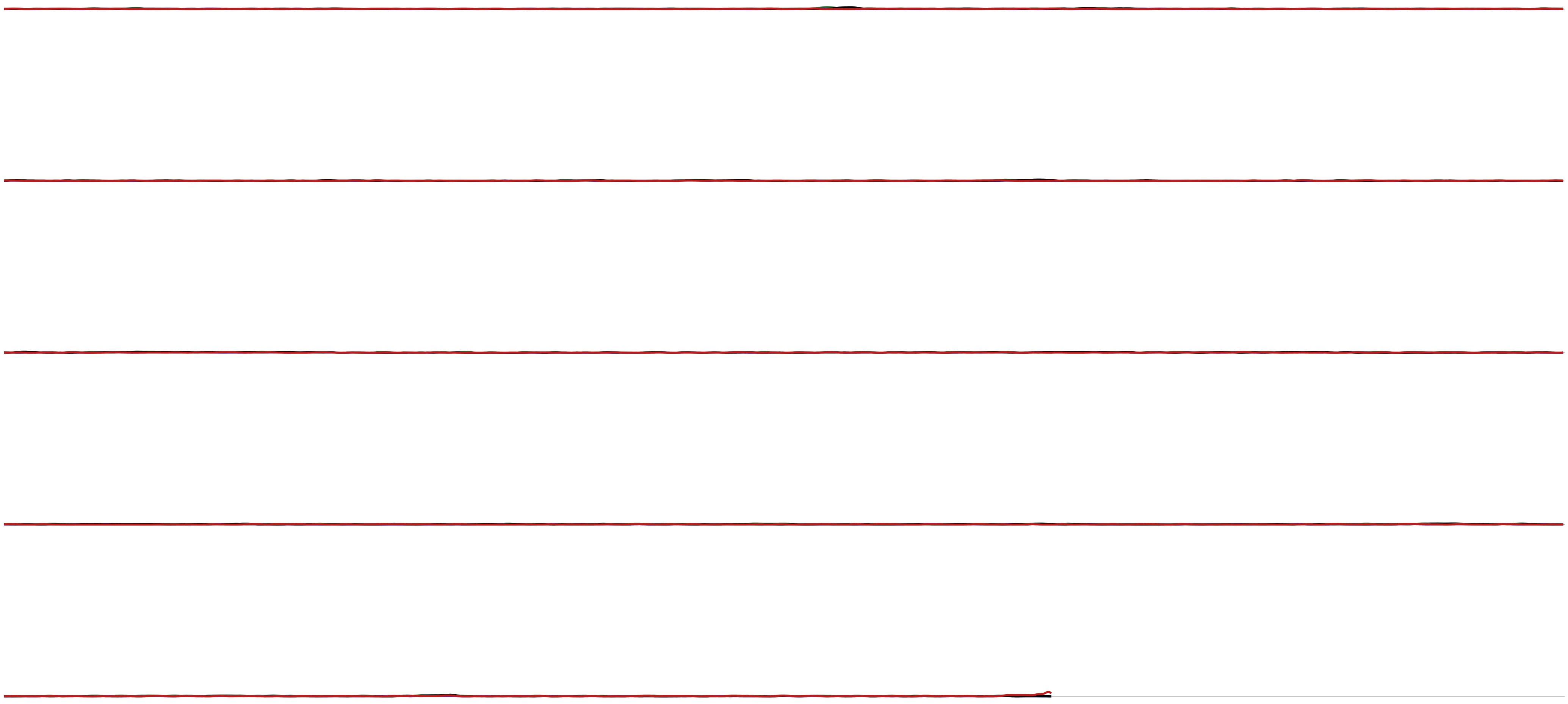

Supplement: Supplemental Information 1 — Sequences for forward and reverse reads from WT and phf21aa mutant fish. These sequences are also found in GenBank, with accession numbers: wild type MW438986 and mutant MW438985. [file peerj-09-11007-s001.zip › phf21aa_Sequencing/Mutant_Reverse.pdf]
