# Supplementary figures and images for: ZebraShare: a new venue for rapid dissemination of zebrafish mutant data
Source: PeerJ. 2021 Apr 13;9:e11007. doi: 10.7717/peerj.11007 (PMC8051354; doi:10.7717/peerj.11007)

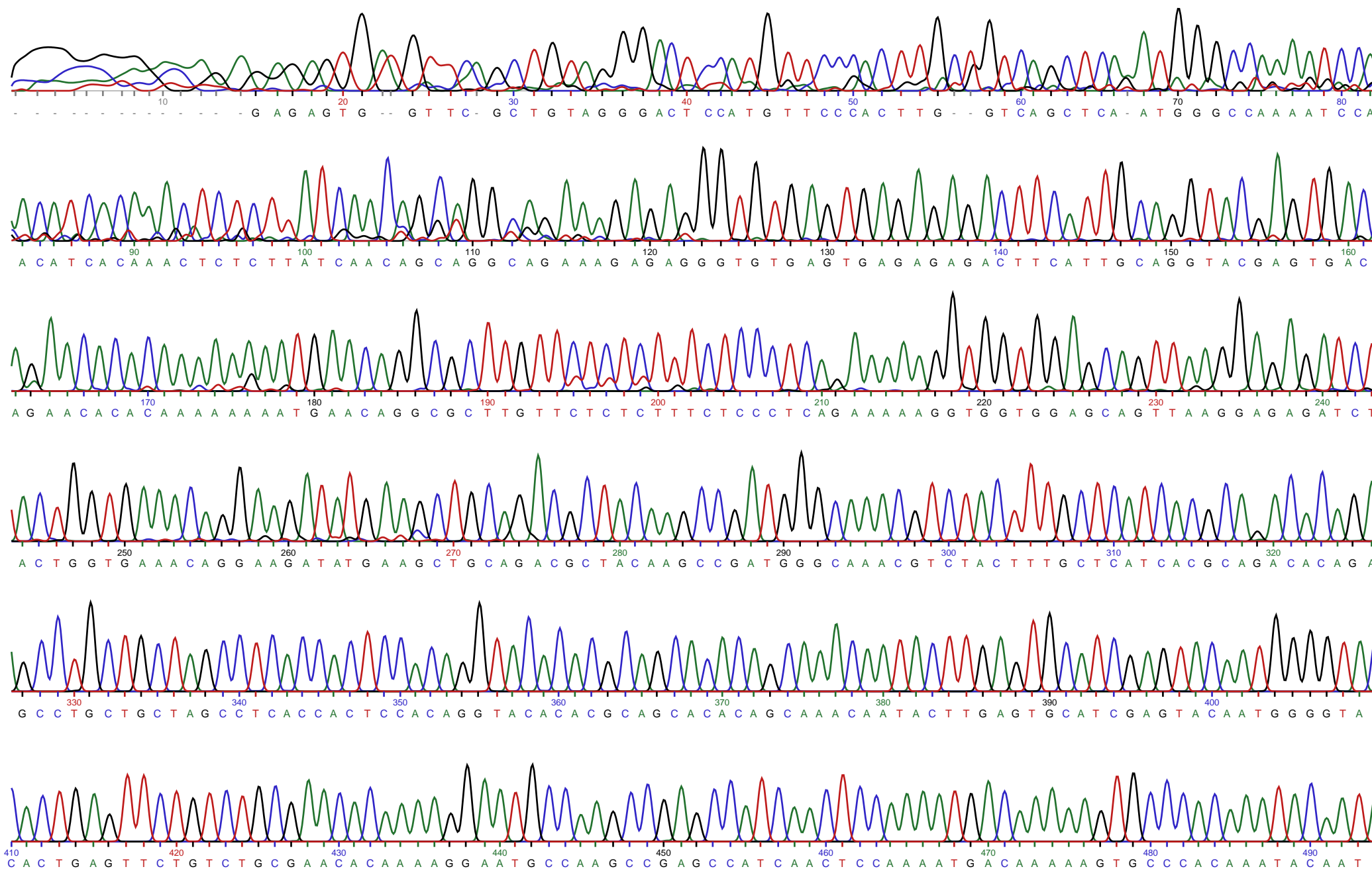

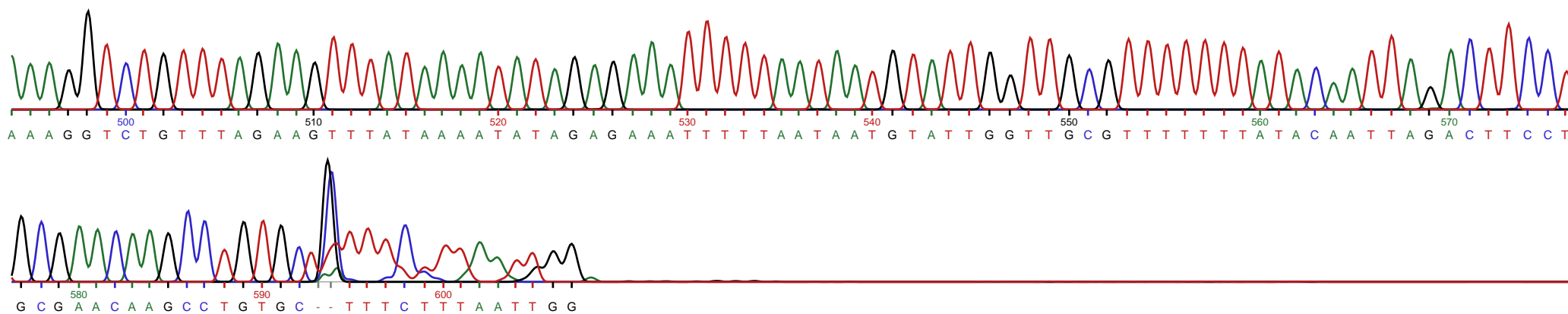

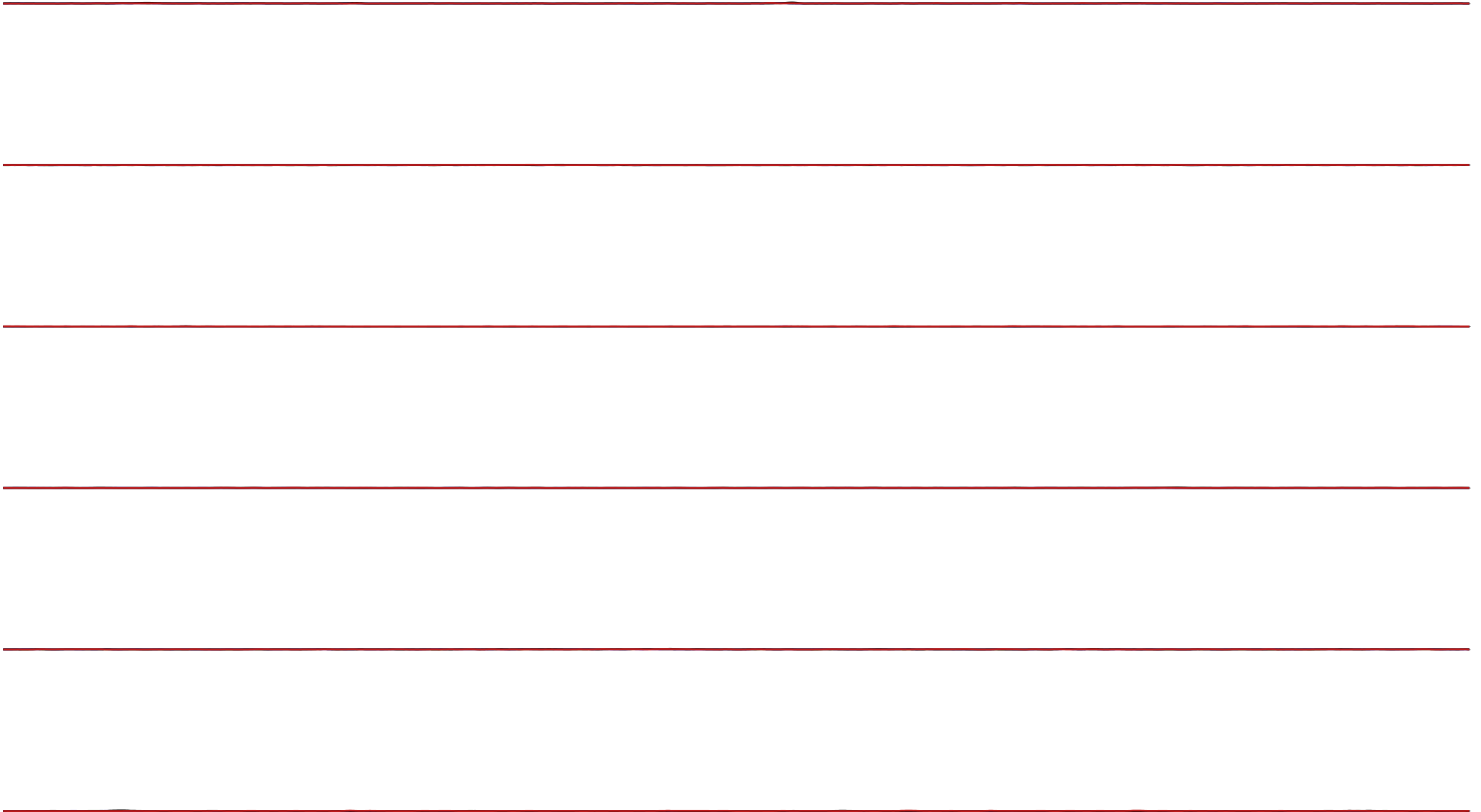

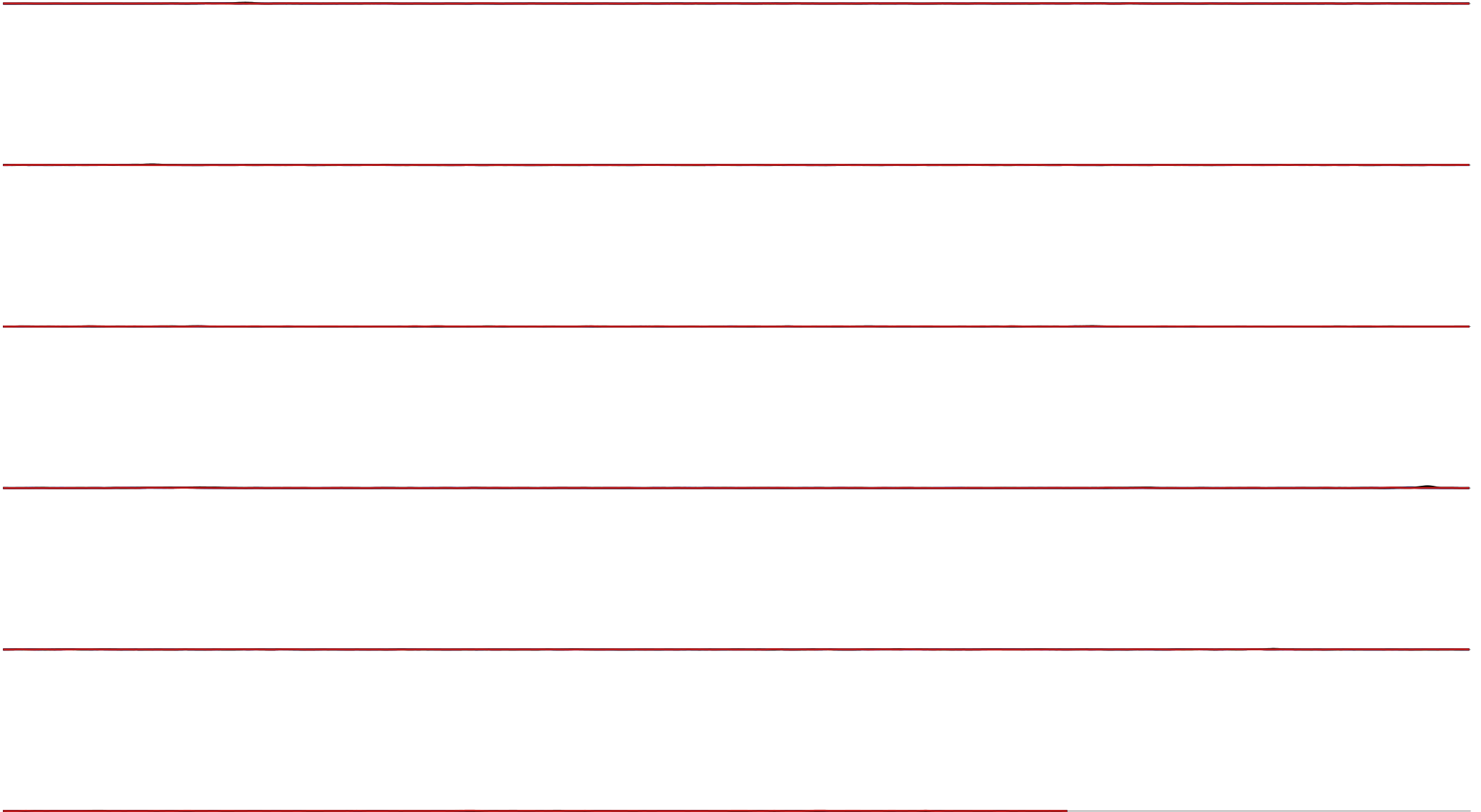

Supplement: Supplemental Information 1 — Sequences for forward and reverse reads from WT and phf21aa mutant fish. These sequences are also found in GenBank, with accession numbers: wild type MW438986 and mutant MW438985. [file peerj-09-11007-s001.zip › phf21aa_Sequencing/Mutant_Forward.pdf]

Samples: 25233  
Bases: 612  
Average spacing: 42

Page: 1 / 5  
8/31/2020

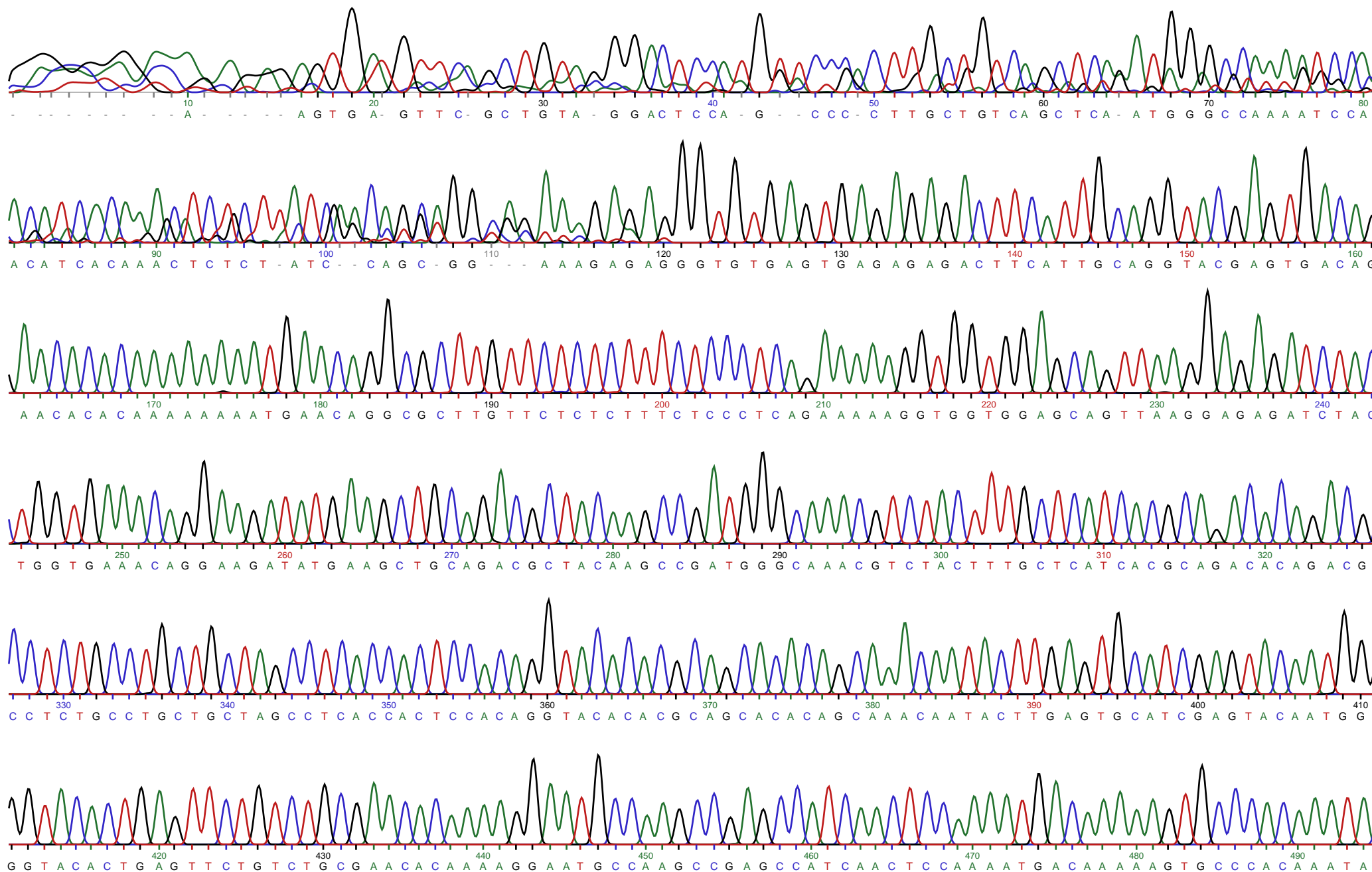

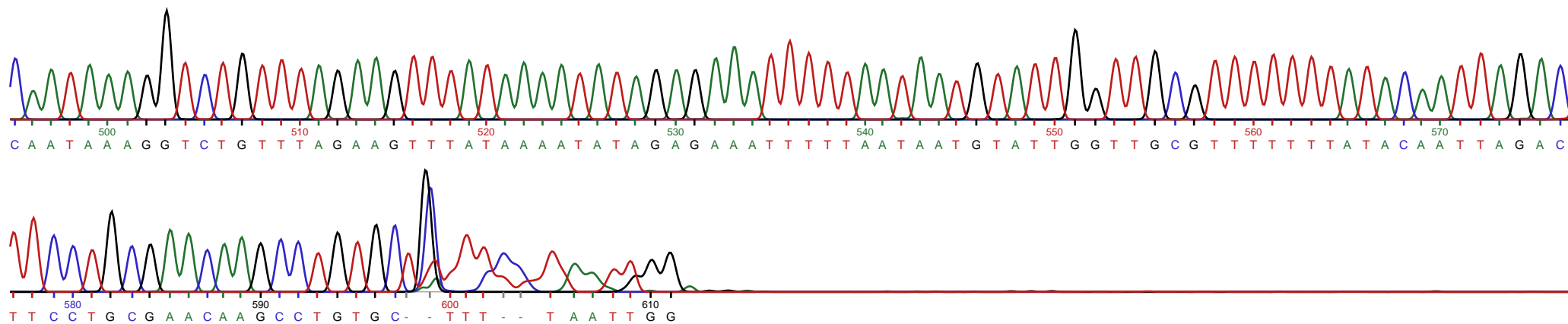

Samples: 25233  
Bases: 612  
Average spacing: 42

Page: 3 / 5  
8/31/2020

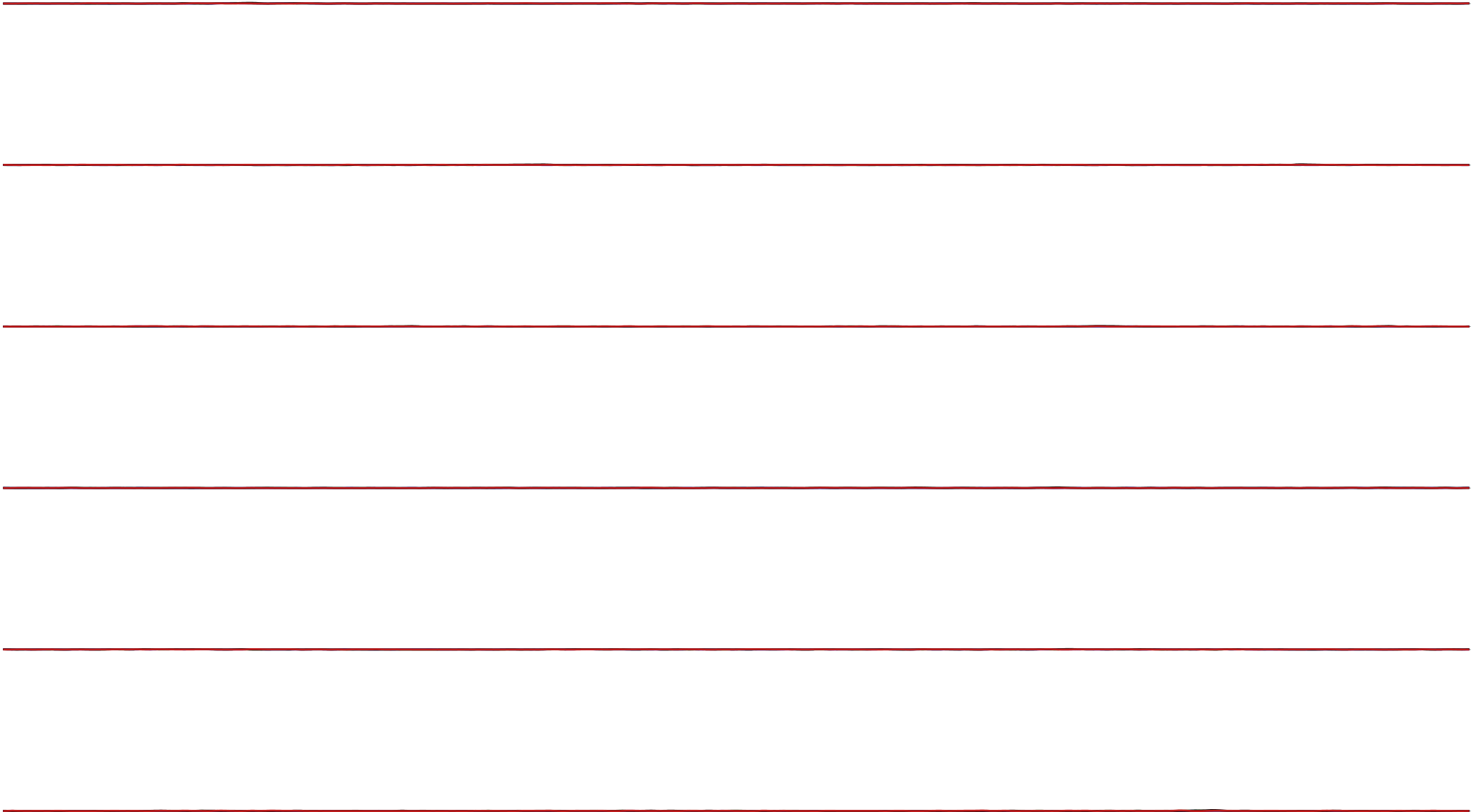

---

---

Supplement: Supplemental Information 1 — Sequences for forward and reverse reads from WT and phf21aa mutant fish. These sequences are also found in GenBank, with accession numbers: wild type MW438986 and mutant MW438985. [file peerj-09-11007-s001.zip › phf21aa_Sequencing/WT_Forward.pdf]

Page: 1 / 4  
8/31/2020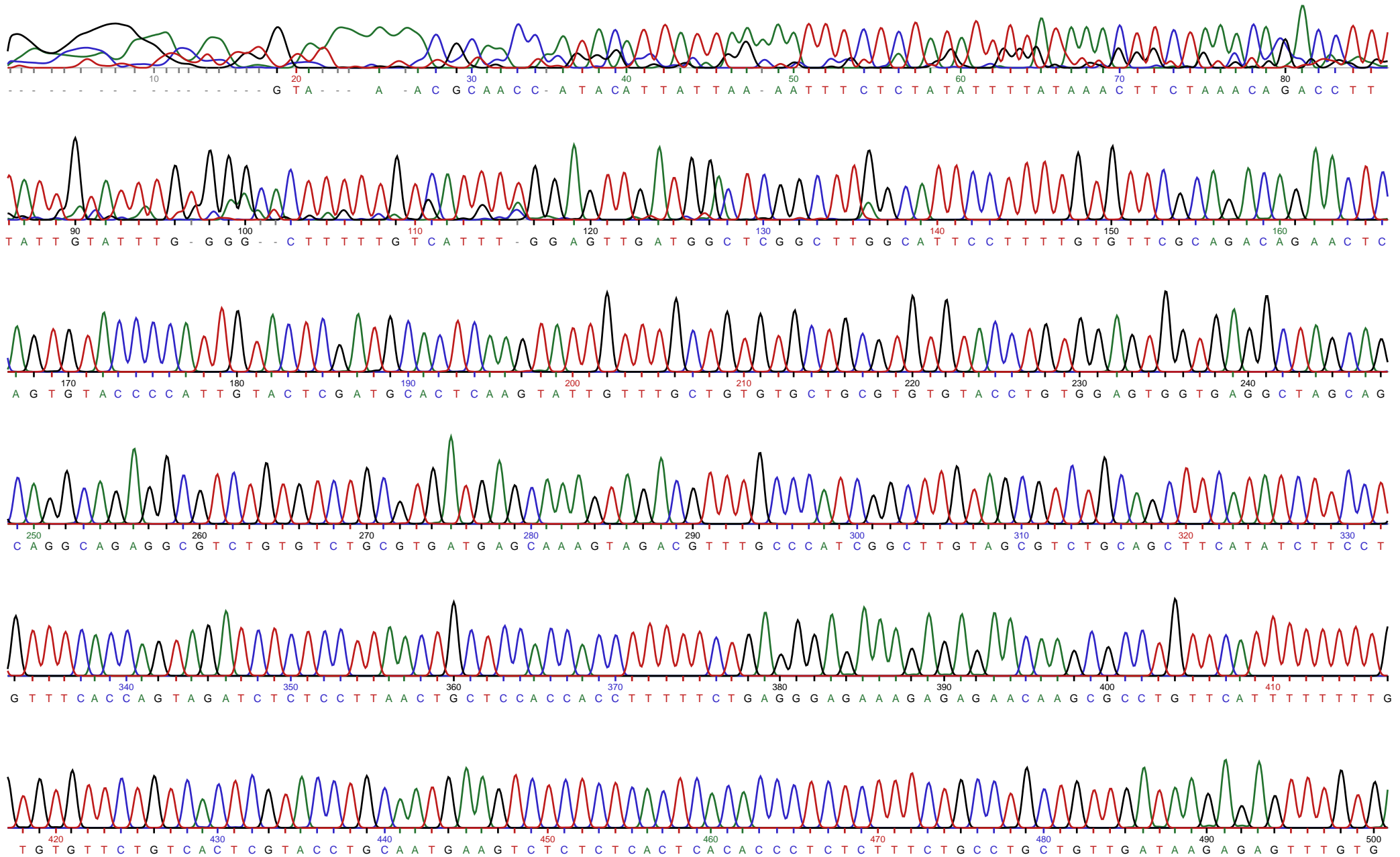

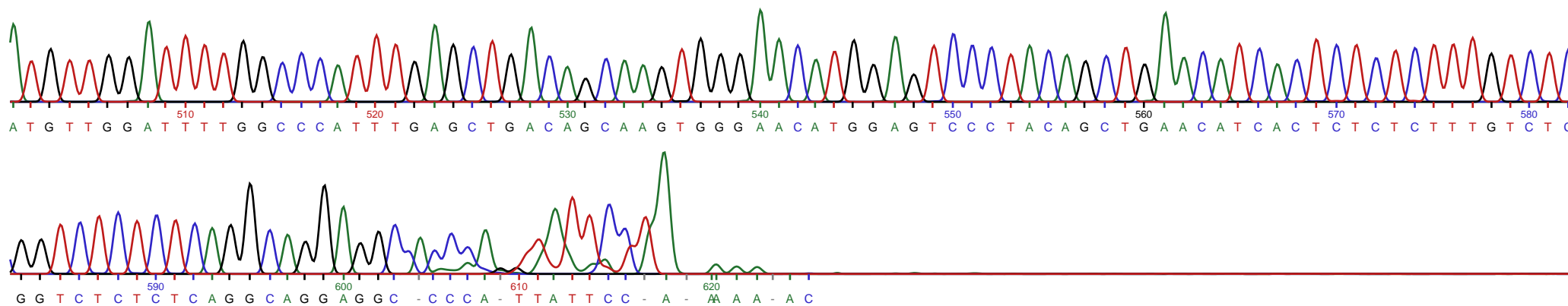

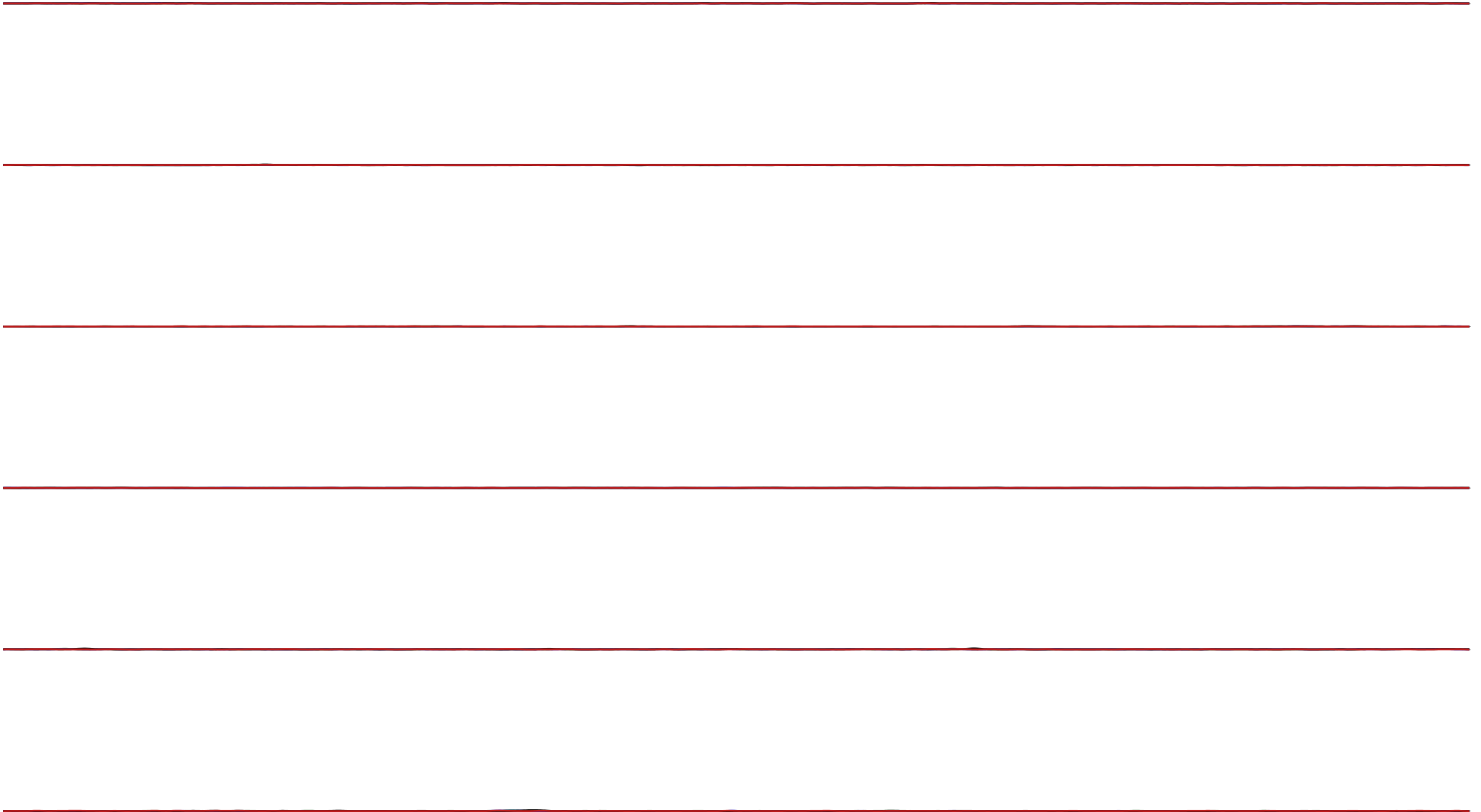

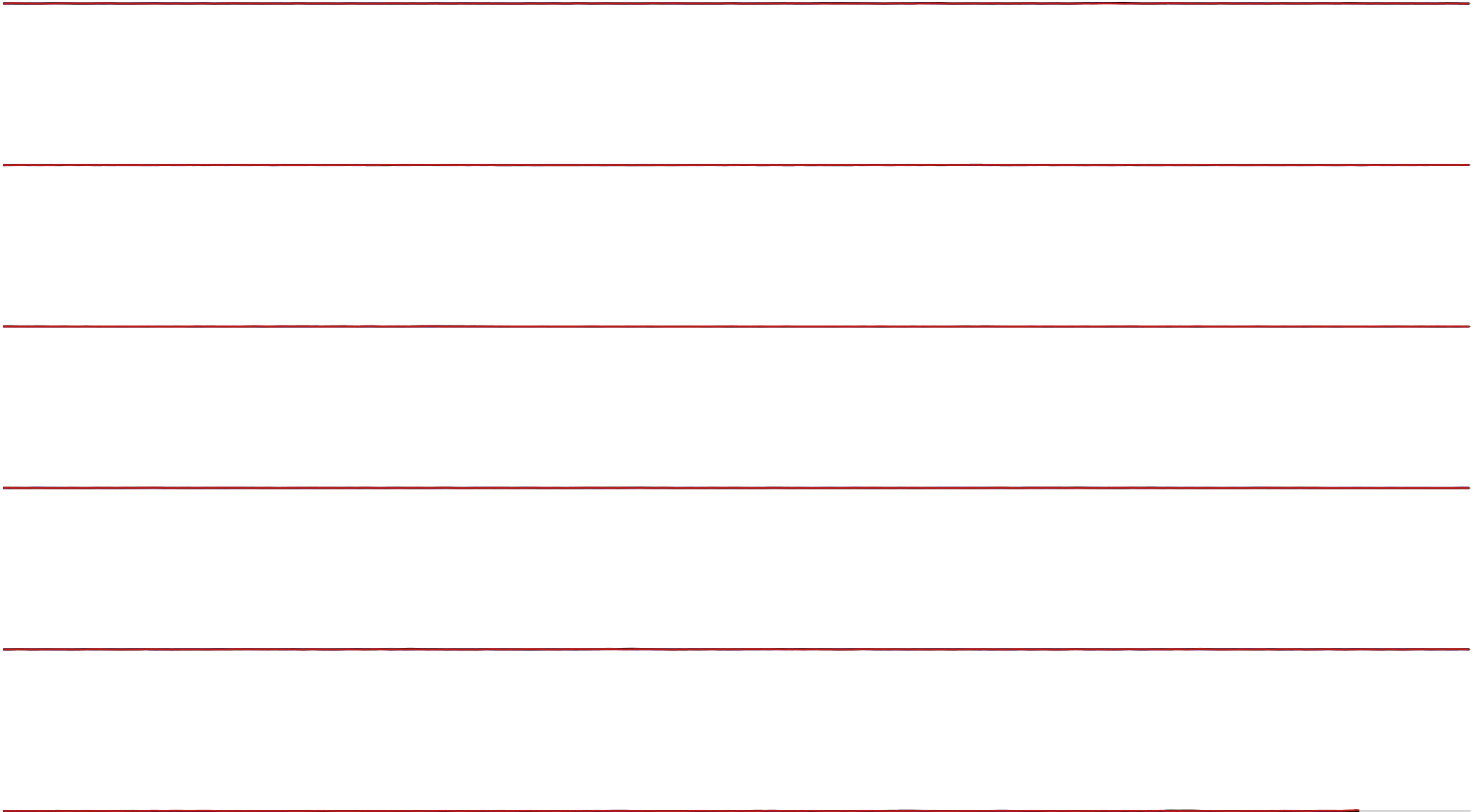

Supplement: Supplemental Information 1 — Sequences for forward and reverse reads from WT and phf21aa mutant fish. These sequences are also found in GenBank, with accession numbers: wild type MW438986 and mutant MW438985. [file peerj-09-11007-s001.zip › phf21aa_Sequencing/WT_Reverse.pdf]
